# Measuring mild cognitive impairment using routinely collected health‐care data: A conceptual framework and guidance for embedded pragmatic clinical trials

**DOI:** 10.1002/alz.71942

**Published:** 2026-11-01

**Authors:** Dae Hyun Kim, Kevin W. McConeghy, Jennifer L. Lund, Ryan Mace, David H. Lynch, Elizabeth M. White, Hua Xu, Rafael Samper‐Ternent, Phillip Wang, Ellen P. McCarthy, NIA IMPACT Collaboratory Technical Data-Health Care Systems Core

**Affiliations:** ^1^ The Hinda and Arthur Marcus Institute for Aging Research, Hebrew SeniorLife, Division of Gerontology Beth Israel Deaconess Medical Center, Harvard Medical School Boston Massachusetts USA; ^2^ Department of Health Services, Policy, and Practice Brown University School of Public Health Providence Rhode Island USA; ^3^ Department of Epidemiology Gillings School of Global Public Health, University of North Carolina at Chapel Hill Chapel Hill North Carolina USA; ^4^ Center for Health Outcomes and Interdisciplinary Research, Department of Psychiatry Massachusetts General Hospital, Harvard Medical School Boston Massachusetts USA; ^5^ Division of Geriatric Medicine University of North Carolina School of Medicine Chapel Hill North Carolina USA; ^6^ Department of Biomedical Informatics and Data Science Yale School of Medicine New Haven Connecticut USA; ^7^ Department of Management, Policy, and Community Health, School of Public Health, Institute on Aging The University of Texas Health Science Center in Houston Houston Texas USA; ^8^ Department of Epidemiology, Harvard T.H. Chan School of Public Health, Division of Pharmacoepidemiology and Pharmacoeconomics, Department of Medicine and Department of Psychiatry Brigham and Women's Hospital Boston Massachusetts USA

**Keywords:** administrative data, electronic health records, informatics, mild cognitive impairment, pragmatic clinical trials

## Abstract

Mild cognitive impairment (MCI) is increasingly measured using routinely collected health‐care data in embedded pragmatic clinical trials. However, accurate ascertainment remains challenging because key diagnostic elements, such as cognitive function and functional status, are often incompletely captured. We identified three broad categories for measuring MCI using routinely collected health‐care data: documentation‐based approaches, which identify clinician‐recognized MCI; clinical criteria‐based approaches, which reconstruct the diagnostic construct using available clinical information; and prediction‐based approaches, which estimate the likelihood of MCI using statistical or machine learning models. These approaches measure distinct constructs and differ in validity and scalability, and susceptibility to bias. For eligibility and outcome ascertainment, clinical criteria‐based approaches should be prioritized whenever feasible. Documentation‐based approaches may serve as complementary measures of clinician‐recognized MCI, whereas prediction‐based approaches are best suited for screening and risk stratification. Continued advances in phenotype development, data standardization, and validation are needed to support accurate and equitable MCI ascertainment.

## Highlights

- This paper proposes a framework for ascertaining mild cognitive impairment (MCI) using routinely collected health‐care data.
- There are three approaches to MCI ascertainment: documentation‐based, clinical‐based, and prediction‐based.
- These ascertainment methods are shown to measure different underlying MCI constructs.
- Practical guidance is provided for measuring MCI in embedded pragmatic clinical trials.
- Priorities are identified for making MCI phenotyping more scalable and equitable.

## 1 BACKGROUND

Mild cognitive impairment (MCI) is a transitional state between normal cognition and dementia, characterized by subjective cognitive concern, objective evidence of impairment in one or more cognitive domains, preserved independence in functional abilities, and absence of another mental disorder such as dementia or delirium.^1^, ^2^, ^3^ MCI affects 15% to 20% of older adults, with prevalence increasing from 6.7% in individuals aged 60 to 64 years to 25.2% in those aged 80 to 84 years.^4^ Individuals with MCI are at increased risk of functional decline,^5^ hospitalization,^6^ and progression to dementia,^7^ and incur more than double the health‐care costs of individuals without MCI or dementia.^8^ These clinical and public health consequences make MCI a critical target for interventions aimed at promoting healthy aging and delaying or preventing dementia.

The need for scalable approaches to MCI ascertainment has increased substantially with the emergence of disease‐modifying therapies for early Alzheimer's disease (AD),^9^, ^10^ expanding cognitive screening initiatives, and growing use of real‐world evidence to evaluate care delivery and outcomes. Embedded pragmatic clinical trials (ePCTs) are increasingly used to test interventions designed to prevent MCI or slow progression to dementia. These trials frequently rely on routinely collected health‐care data, such as electronic health records (EHRs) and administrative claims data, to enable scalable recruitment and outcome ascertainment.

However, MCI is inherently challenging to identify using routinely collected health‐care data. Diagnosis requires objective cognitive testing, documentation of functional independence, and exclusion of dementia and delirium—elements that are not systematically or consistently captured and often under‐coded in structured EHR fields or administrative claims. Cognitive screening is performed variably across care settings, test results are frequently missing or recorded in unstructured formats, and functional status, including activities of daily living (ADLs) and instrumental activities of daily living (IADLs), are infrequently documented. Consequently, ascertainment of MCI based solely on International Classification of Diseases (ICD) diagnosis codes captures only a small fraction of cases,^11^, ^12^, ^13^ leading to potential misclassification and biased estimates in research using routinely collected health‐care data.

This paper provides an overview of existing and emerging approaches for measuring MCI using routinely collected health‐care data and outlines key design considerations for MCI measurement in ePCTs. Although focused on ePCTs, many of the challenges and approaches discussed are broadly relevant to observational studies, comparative effectiveness research, quality measurement, and implementation of dementia care programs using routinely collected health‐care data.

## 2 GENERAL CONSIDERATIONS FOR MEASURING MCI USING ROUTINELY COLLECTED HEALTH‐CARE DATA IN ePCTs

The desired properties for MCI measurement differ depending on the role of MCI within an ePCT—whether used as an eligibility criterion or as an outcome—necessitating careful alignment between study objectives and measurement strategies. When MCI is used as an *eligibility criterion*, the optimal measurement properties depend on the enrollment strategy. For initial screening, approaches often prioritize higher sensitivity to identify a broader pool of potentially eligible individuals, accepting some false positives that can subsequently be excluded through confirmatory clinical assessment, which typically involves structured cognitive and functional evaluation adjudicated against established clinical criteria^1^, ^2^, ^3^ (we acknowledge that no biological confirmatory test of MCI currently exists). However, in many ePCTs, confirmatory cognitive evaluation may be impractical because of cost, scale, or workflow constraints. In such settings, higher specificity and positive predictive value become more important to ensure that enrolled participants truly have MCI. In contrast, when MCI is used as an outcome, accurate and unbiased classification is essential for valid estimation of intervention effects (on the relative scale) and approaches generally prioritize higher specificity and positive predictive value to minimize false positives.^14^ Importantly, whether MCI is used as an eligibility criterion or an outcome, MCI must be ascertained using the same methods in both intervention and control groups.

The EHR data‐generating process—routine clinical assessment and documentation by care providers—introduces variability in how MCI is identified and recorded. Because clinically recognized MCI is under‐coded,^15^, ^16^ diagnosis‐based ascertainment alone lacks sensitivity. The richness of EHR data allows development of composite phenotypes that integrate diagnostic codes, health‐care use patterns, medications, and unstructured clinical text.^17^, ^18^ While these approaches may improve case identification, they require substantial effort and access to reference‐standard clinical data for model development and validation.^19^ Probabilistic models may estimate the likelihood of MCI based on the patient's demographic and clinical characteristics. However, their performance requires a reliable case definition and will depend on the quality and completeness of the data.

MCI ascertainment also varies by care setting, access to diagnostic evaluation, and sociodemographic factors.^20^ Inconsistent or infrequent health‐care access may lead to delayed or missed recognition of MCI, resulting in differential ascertainment across populations. In addition, the lag between symptom onset and clinical documentation can result in reverse causation when MCI is documented after intervention initiation. Investigators should therefore consider the potential for differential ascertainment and misclassification when selecting and interpreting MCI measures in ePCTs. These issues, including the implications of health‐care disparities for MCI ascertainment, are discussed in greater detail later.

Distinguishing stable MCI from MCI due to AD has direct implications for ePCT eligibility and outcome ascertainment. Anti‐amyloid disease‐modifying therapies require biomarker‐confirmed MCI due to AD, whereas routinely collected health‐care data may not always document the etiology. When etiological specificity is required, investigators should supplement routinely collected health‐care data with biomarker confirmation, recognizing this may reduce eligible sample size and generalizability given limited biomarker access outside specialist settings.

## 3 ROUTINE DATA SOURCES AND THEIR MEASUREMENT IMPLICATIONS

Two primary types of routinely collected health‐care data in the United States are used to identify MCI: administrative claims data and EHR data. Because these sources differ in structure, purpose, and data‐generating processes, they offer distinct strengths and limitations for MCI ascertainment.

### 3.1 Administrative claims data

Claims data are generated through billing and reimbursement processes and capture information on enrollment, diagnoses, procedures, medication dispensing, and amounts charged or reimbursed. Diagnoses are recorded using ICD‐10 diagnosis codes; procedures in ICD‐10 procedures codes and Current Procedural Terminology codes; medications in National Drug Codes. Provider specialty codes enable identification of encounters with clinicians involved in cognitive evaluation (e.g., neurology, psychiatry, geriatrics), although the completeness varies across insurance types (e.g., Medicare fee‐for‐service, Medicare Advantage, Medicaid, commercial insurance).

#### 3.1.1 Strengths

Claims data are highly scalable, standardized, and support longitudinal follow‐up across care settings and large populations. Linkage with assessment datasets such as the Minimum Data Set (MDS) and Outcome and Assessment Information Set (OASIS) can capture information on cognitive and functional status.

#### 3.1.2 Limitations

Because claims data are designed for billing rather than clinical phenotyping, they generally lack detailed cognitive, functional, and neuropsychological information required for MCI diagnosis. Ascertainment may also vary across health‐care systems, insurance types, and populations because of differences in eligibility, coding practices, health‐care access, and data completeness.

### 3.2 EHR data

EHR data are used to document clinical care and contain both structured information (e.g., problem list, diagnoses, procedures, medications, orders, and laboratory results) and unstructured information (e.g., cognitive symptoms, functional status, imaging reports, clinical impressions). Extraction of unstructured data requires natural language processing and related analytic methods.^21^, ^22^

#### 3.2.1 Strengths

EHR data provide richer clinical information than claims data and may capture cognitive symptoms, functional status, clinical assessments, and test results relevant to MCI diagnosis. They also enable integration of multimodal information across clinical domains.

#### 3.2.2 Limitations

Documentation of cognitive and neuropsychological testing and functional status is often incomplete, inconsistent, or embedded in scanned documents or free‐text notes.^22^ Longitudinal follow‐up may also be limited when patients receive care across multiple health‐care systems. Variability across care settings, health‐care organizations, EHR platforms, and insurance coverage further limits completeness, generalizability, and scalability.

The strengths and limitations of claims and EHR data shape the feasibility and performance of the three MCI ascertainment approaches described below.

## 4 APPROACHES FOR MEASURING MCI IN ROUTINELY COLLECTED HEALTH‐CARE DATA

To characterize existing approaches for identifying MCI using routinely collected health‐care data, we searched PubMed (medical subject heading terms and title/abstract fields) from January 1, 2000, through May 31, 2026, combining terms for MCI with terms for EHR and administrative claims, restricted to English‐language publications. Although individual algorithms vary in data sources, predictors, and analytic methods, most can be grouped into three broad categories based on the underlying strategy used to ascertain MCI (Figure 1): (1) identifying clinician‐recognized MCI (documentation‐based approach), (2) reconstructing the diagnostic construct (clinical criteria‐based approach), and (3) estimating the probability of MCI (prediction‐based approach).

**FIGURE 1:**
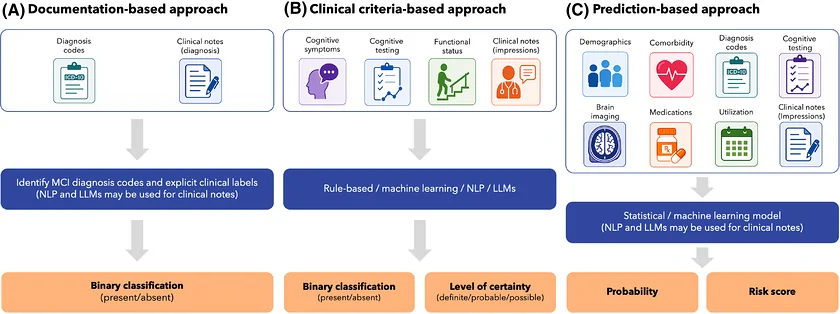
Approaches for measuring mild cognitive impairment using routinely collected health‐care data. This figure illustrates three complementary approaches to identifying MCI from routinely collected health‐care data. (A), The documentation‐based approach identifies cases based on clinician‐recorded diagnoses (e.g., diagnosis codes or documented labels) and produces a binary classification. (B), The clinical criteria–based approach approximates the clinical diagnostic construct of MCI by integrating cognitive symptoms, testing, functional status, and clinical impressions, generating algorithm‐defined classifications or levels of diagnostic certainty. (C), The prediction‐based approach estimates the likelihood of current or future MCI using statistical or machine learning models applied to a broad set of predictors, producing a continuous probability or risk score. These approaches differ in their roles: documentation‐based and clinical criteria‐based approaches define MCI, whereas prediction models support identification of individuals at higher likelihood of MCI but do not define MCI. Accordingly, they are suited to different purposes in embedded pragmatic clinical trials. LLM, large language model; MCI, mild cognitive impairment; NLP, natural language processing.

These approaches measure related but distinct constructs and should not be considered interchangeable. Documentation‐based approaches measure clinician‐recognized MCI, clinical criteria‐based approaches approximate the underlying diagnostic construct, and prediction‐based approaches estimate the probability of MCI. As a result, they differ in their outputs, performance characteristics, and suitability for specific research and clinical applications. Table 1 summarizes representative studies identified through the literature review.

**TABLE 1:** Mild cognitive impairment algorithms using routinely collected health‐care data^*^.

Author	Data source and population	MCI identification approach	Performance
**Documentation‐based approach**			
McCarthy (2022)^16^	HRS‐Medicare linked data from 5784 participants, 2012	Any presence of ICD codes	Outcome: CIND based on cognitive assessment or proxy report Sensitivity: 0.03 Specificity: 1.00 PPV: 0.18 NPV: 0.82
		Bynum algorithm based on ICD codes	Outcome: CIND or dementia based on cognitive assessment or proxy report Sensitivity: 0.17 Specificity: 0.99 PPV: 0.89 NPV: 0.74
Aguilar (2023)^11^	EHR data from 339,007 veterans, 2010–2019	Keyword search “mild cognitive impairment” in clinical notes	Outcome: MCI from manual review of clinical notes PPV: 0.97
Kistler (2026)^23^	EHR data from 525 patients in primary care clinics, North Carolina, 2021–2022	Any presence of ICD codes for cognitive impairment	Outcome: MCI or dementia from manual review of clinical notes Sensitivity: 0.60 Specificity: 0.98 PPV: 0.75 NPV: 0.96 F1: 0.67
**Clinical criteria‐based approach**			
Wei (2025)^19^	EHR data from 3626 outpatients at two academic hospitals in Massachusetts, 2015–2022	Lasso logistic regression Random forest Naïve Bayes Multi‐layer perceptron using ICD codes, medications, and clinical notes	Outcome: MCI or dementia from expert review of clinical notes Sensitivity: 0.91 Specificity: 0.96 PPV: 0.96 AUROC: 0.98 AUPRC: 0.98 F1: 0.93
**Prediction‐based approach**			
Wang (2021)^28^	EHR clinical notes from 2166 patients with MCI diagnosis at two academic hospitals in Massachusetts, 2015–2019	Deep learning Logistic regression Random forest Support vector machine XGBoost based on clinical notes	Outcome: Any stage of cognitive decline documented in clinical notes Sensitivity: 0.93 PPV: 0.77 AUROC: 1.00 AUPRC: 0.93
Bacigalupo (2022)^15^	Administrative data from 2608 patients in 24 geriatrics or neurology clinics, Italy, 2012–2016	Logistic regression based on ICD codes, medications, co‐payment exemption, long‐term care records, age, sex	Outcome: MCI based on clinical criteria Sensitivity: 0.30 Specificity: 0.98 PPV: 0.92 NPV: 0.58 AUROC: 0.64
			Outcome: MCI or dementia based on clinical criteria Sensitivity: 0.68 Specificity: 0.96 PPV: 0.95 NPV: 0.75 AUROC: 0.82
Penfold (2022)^27^	EHR data from 1794 participants in the ACT study and 2391 patients of Kaiser Permanente Washington, 2004–2015	Lasso logistic regression based on sociodemographic factors, cognitive and behavioral symptoms from clinical notes	Outcome: MCI defined by MMSE and MoCA Sensitivity: 0.02 Specificity: 1.00 PPV: 0.70 NPV: 0.70 AUROC: 0.67 F1: 0.04
Du (2024)^29^	EHR data from 2166 patients with MCI diagnosis at two academic hospitals in Massachusetts, 2015–2019	Ensemble deep learning and large language model using clinical notes	Outcome: Any stage of cognitive decline documented in clinical notes Sensitivity: 1.00 Specificity: 1.00 PPV: 1.00 NPV: 1.00 F1: 1.00
Floden (2025)^26^	EHR data from 337 primary care patients, Cleveland, 2020–2022	Logistic regression based on age, race, pulse, systolic blood pressure, non‐steroidal anti‐inflammatory use, history of mood disorder, and family history of neurological disease from EHR	Outcome: MCI based on neuropsychological testing Sensitivity: 0.38 Specificity: 0.93 PPV: 0.75 NPV: 0.77 AUROC: 0.72
Li (2025)^18^	Optum Clinical EHR database including 285555 patients, 2014–2022	Bayesian lasso logistic regression based on demographic factors, symptoms, signs, and diseases from semi‐structured EHR data	Outcome: MCI ICD codes Sensitivity: 0.78 Specificity: 0.80 AUROC: 0.86
Leng (2025)^30^	EHR clinical notes from 814 Medicare beneficiaries, 2016–2018	Large language model using clinical notes	Outcome: Cognitive impairment stage (unimpaired, MCI, dementia) based on clinician adjudication Weighted Cohen κ = 0.95 Spearman correlation = 0.93

Abbreviations: ACT, Adult Changes in Thought Study; AUPRC, area under the precision‐recall curve; AUROC, area under the receiver operating characteristics curve; CIND, cognitive impairment–no dementia; EHR, electronic health record; HRS, Health and Retirement Study; ICD, International Classification of Diseases; MCI, mild cognitive impairment; MMSE, Mini‐Mental State Examination; MoCA, Montreal Cognitive Assessment; NPV, negative predictive value PPV, positive predictive value.

* Only studies that investigated MCI or MCI/dementia were included. Studies that exclusively examined dementia were excluded from the table. Performance statistics from the testing set are reported.

### 4.1 Documentation‐based approach

The documentation‐based approach identifies MCI based on clinician‐recognized and documented diagnoses in routinely collected health‐care data. Individuals are classified as having MCI if clinicians explicitly document the condition using structured data, such as diagnosis codes, problem lists, billing codes, or if such documentation can be identified from unstructured clinical notes. The defining feature of this approach is that MCI ascertainment depends on the presence of an explicit diagnostic label or clinical impression.

Implementation may involve direct extraction of diagnosis codes or terms from clinical records. For example, McCarthy et al.^16^ and Kistler et al.^23^ used ICD‐based algorithms. Diagnosis codes may include specific MCI diagnosis codes as well as non‐specific codes for cognitive symptoms (Table 2). Aguilar et al. conducted targeted searches for the term “mild cognitive impairment” in unstructured notes.^11^ Natural language processing or machine learning may be used to extract or classify clinician‐documented diagnoses from unstructured text. Regardless of method, the outcome reflects documented MCI.

**TABLE 2:** List of International Classification of Diseases 10th revision diagnosis codes for mild cognitive impairment and related symptoms.

Codes	Description
**Specific codes for mild cognitive impairment**	
F06.70	Mild neurocognitive disorder due to a known physiological condition, with behavioral disturbance
F06.71	Mild neurocognitive disorder due to a known physiological condition, without behavioral disturbance
G31.84	Mild cognitive impairment of uncertain or unknown etiology
**Non‐specific codes for cognitive symptoms and diagnoses (other than dementia)**	
R41.0	Disorientation, unspecified
R41.1	Anterograde amnesia
R41.2	Retrograde amnesia
R41.3	Other amnesia
R41.4	Neurologic neglect syndrome
R41.81	Age‐related cognitive decline
R41.82	Altered mental status, unspecified
R41.84	Cognitive communication deficit
R41.89	Other symptoms and signs involving cognitive functions and awareness
R41.9	Unspecified symptoms and signs involving cognitive functions and awareness

Documentation‐based approaches are highly scalable and often have high specificity when based on structured diagnoses or problem lists. However, they generally have low sensitivity because many individuals with MCI, particularly those with mild or early impairment, remain undiagnosed or undocumented in routine care. Specificity may also be lower when MCI is identified from free‐text documentation, when the term may be used descriptively or inconsistently, including among individuals with dementia or non‐specific cognitive symptoms. Because ascertainment depends on clinical recognition and documentation, estimates derived from these approaches are influenced by health‐care access, use patterns, clinician practices, and coding behavior.

### 4.2 Clinical criteria‐based approach

The clinical criteria‐based approach seeks to approximate the clinical diagnostic construct of MCI using information available in routinely collected health‐care data. Rather than relying on an explicitly documented MCI diagnosis, this approach uses data elements reflecting components of diagnosis—such as cognitive impairment, functional status, symptoms, and clinical assessment—to approximate whether an individual meets criteria for MCI, even in the absence of a formal diagnosis. In contrast to documentation‐based approaches, MCI ascertainment is based on reconstruction of the diagnostic construct rather than identification of a recorded diagnosis. In this approach, the outcome is defined by clinical criteria, such as cognitive test results, symptoms, functional status, or adjudicated clinical assessment, and the predictors are selected because they reflect domains that are integral to diagnosis. For example, Wei et al. used information extracted from clinical notes to mimic expert clinical assessment.^19^

Implementation may involve rule‐based algorithms or machine learning methods, including natural language processing, to extract and synthesize information from unstructured notes. However, performance depends on the availability and quality of relevant clinical information. When cognitive function, symptoms, functional status, and clinician's impressions are infrequently documented, particularly before formal diagnosis, natural language processing may not reliably support early detection.^24^

Clinical criteria‐based approaches produce binary classifications (present or absent) or levels of diagnostic certainty (definite, probable, possible MCI). Compared to documentation‐based approaches, they can identify a broader group of individuals with MCI and more closely reflect the underlying clinical condition. However, they generally require richer clinical data and more complex implementation, and their performance may vary across health‐care settings because of differences in documentation practices and data availability. Commonly used cognitive screening instruments (e.g., the Mini‐Mental State Examination and Montreal Cognitive Assessment) are frequently incorporated into clinical criteria‐based algorithms and used as eligibility thresholds in clinical trials. The field lacks consensus on cutoff scores distinguishing MCI from normal cognition or early dementia, and reported optimal cutoffs vary by population, age, and education.^25^ Assessments of ADLs and IADLs are similarly central, because preserved functional independence defines MCI relative to dementia, but they may miss the subtler functional changes typical of MCI (e.g., difficulty with complex financial tasks).

### 4.3 Prediction‐based approach

The prediction‐based approach estimates the likelihood of current or future MCI using statistical or machine learning models applied to a broad set of predictors. These predictors may include demographic characteristics, comorbidities, medications, health‐care use, and information derived from clinical notes, and is not required to correspond to components of the MCI diagnostic criteria.

In this approach, MCI definitions, typically based on diagnosis codes or clinical assessments, are used to train models. However, the model output is a probability or risk score, rather than a defined MCI phenotype. Unlike clinical criteria‐based approaches, prediction‐based approaches do not attempt to reconstruct the diagnostic construct of MCI.

Several studies have used structured EHR or claims data. Li et al. generated individualized risk estimates to identify those with a higher likelihood of having MCI diagnosis codes.^18^ Bacigalupo et al. used administrative claims data to predict MCI defined by a consensus diagnostic framework,^15^ while Floden et al. developed an EHR‐based model using seven variables to predict MCI defined by neuropsychological testing.^26^ Other studies incorporated information from unstructured clinical notes. Penfold et al. used sociodemographic characteristics and cognitive and behavioral symptoms extracted from clinical notes to predict MCI defined by cognitive testing.^27^ More recently, Wang et al.,^28^ Du et al.,^29^ and Leng et al.^30^ applied deep learning and large language models to identify cognitive decline or cognitive impairment from unstructured clinical notes. These approaches relied on patterns in health‐care data and clinical documentation rather than direct assessment of MCI diagnostic criteria.

Prediction‐based approaches are useful for screening and risk stratification, particularly when direct clinical information is incomplete. However, because they do not explicitly define MCI as a clinical or diagnostic outcome, they are not interchangeable with documentation‐based or clinical criteria‐based approaches. Their outputs represent likelihood rather than ascertainment and may be influenced by underlying health‐care use patterns, data availability, and model specification.

Taken together, these approaches represent fundamentally different strategies for measuring MCI in routinely collected health‐care data. Documentation‐based approaches maximize scalability and identify clinician‐recognized MCI but substantially under‐ascertain cases. Clinical criteria‐based approaches most closely approximate the diagnostic construct of MCI and can identify individuals who have not yet received a formal diagnosis, but they require richer clinical data and more complex implementation. Prediction‐based approaches are useful for screening and risk stratification when direct clinical information is incomplete, but they generate estimates of likelihood rather than MCI phenotypes. Consequently, the choice of approach should be guided by the study objective, available data sources, and whether the goal is to identify documented MCI, approximate the clinical diagnosis, or estimate risk.

## 5 RECOMMENDED MEASUREMENT APPROACHES FOR ePCTs

Measurement strategies should be selected based on whether MCI is used for an eligibility criterion or as an outcome in ePCT.^31^, ^32^ Given the underdiagnosis and structural barriers to identifying MCI in routine care,^16^, ^23^, ^33^, ^34^, ^35^, ^36^ no single approach is sufficient. Investigators should consider complementary strategies and sensitivity analyses to assess robustness.

### 5.1 MCI as an eligibility criterion

When MCI is used as an eligibility criterion, the goal is to identify individuals at risk for dementia or those most likely to benefit from intervention.^37^, ^38^ In this setting, sensitivity and inclusiveness are prioritized, particularly if confirmatory clinical assessment is planned, and some false‐positive misclassification is acceptable. Eligibility is therefore best conceptualized as risk‐based rather than diagnostic,^39^, ^40^ as many individuals with MCI remain undiagnosed in routine care.^41^, ^42^, ^43^ When confirmatory clinical assessment is impractical, specificity and positive predictive value are increasingly prioritized. The same approach should be used to identify and enroll eligible participants in both the intervention and control groups.

#### 5.1.1 Recommended approaches

Clinical criteria‐based approaches are preferred because they can identify individuals beyond those with documented diagnoses. Approaches that incorporate clinical‐domain information, such as cognitive symptoms, test results, functional status, and information from clinical notes, can improve sensitivity and better reflect the clinical construct.

Prediction‐based approaches are well suited for screening, particularly when direct clinical data are incomplete. These models can efficiently identify individuals at higher likelihood of MCI. In practice, a two‐stage strategy is often appropriate: initial identification using EHR‐based criteria‐based methods, followed by targeted clinical assessment when confirmation is required for enrollment.

#### 5.1.2 Less suitable approaches

Documentation‐based approaches alone are not suitable for eligibility, as they rely on clinician recognition and substantially under‐ascertain individuals with undiagnosed or milder MCI. As a result, these approaches may preferentially identify individuals who are more clinically recognized, symptomatic, engaged with health care, or evaluated by specialists, while excluding those with earlier or unrecognized MCI. This may reduce the sample size and generalizability.

### 5.2 MCI as a trial outcome

When MCI is used as an ePCT outcome, the goal is to ascertain MCI or its progression to dementia consistently across treatment arms.^44^, ^45^ In this setting, specificity, clinical interpretability, and minimizing differential misclassification are prioritized. Defining MCI as a trial outcome also requires specifying tolerance for false negatives and false positives, as asymmetric misclassification can differentially attenuate estimated treatment effects, and aligning ascertainment windows with intervention timing to avoid immortal time bias and reverse causation. We note that “MCI as an outcome” here means MCI incidence, progression, or diagnostic status as a discrete ePCT endpoint—distinct from the broader set of trial outcomes (cognition, function, quality of life, caregiver burden) evaluated once an MCI population has been identified.^46^

#### 5.2.1 Recommended approaches

Clinical criteria‐based approaches are preferred for defining outcomes, particularly those anchored to clinical assessments or multimodal EHR data that approximate the diagnostic construct of MCI. Within this approach, tiered diagnostic‐certainty definitions (e.g., definite, probable, possible MCI) are conceptually appealing because they may enhance interpretability and allow sensitivity analyses across levels of certainty. However, we are not aware of empirical validation of this specific tiering scheme, and it should be treated as exploratory pending such validation.

Documentation‐based approaches may also be used when the outcome is explicitly defined as “documented MCI,” recognizing that this reflects clinical recognition rather than true disease incidence. These approaches are scalable and specific but may be influenced by health‐care use and documentation patterns. Sensitivity analyses using alternative definitions are recommended.

#### 5.2.2 Less suitable approaches

Prediction‐based approaches are less appropriate as primary outcomes, as they do not define MCI as a clinical or diagnostic endpoint. Their outputs represent probabilities rather than ascertainment and may be influenced by patterns of health‐care use and data availability.

### 5.3 Reporting and transparency standards

Given the limitations of MCI measurement in routinely collected health‐care data, transparent reporting is essential.^39^, ^47^ Investigators should describe data sources, algorithms, code lists, observation windows, inclusion and exclusion criteria, and methods to extract information from unstructured data. The construct being measured should be explicitly defined (e.g., “documented MCI,” “clinical criteria‐based MCI,” or “predicted risk of MCI”) to avoid overstating diagnostic certainty. Where available, validation studies and performance characteristics should be reported to support interpretation. Because coding practices, clinical documentation, and terminology used in unstructured notes may vary across health‐care systems and settings, investigators should also consider local pilot validation or targeted chart review to assess whether ascertainment methods identify the intended population and perform consistently across sites. Clear reporting is necessary to support transparency, reproducibility, comparison across studies, and translation into real‐world care.

Based on the considerations outlined above, Table 3 provides practical recommendations for ePCT investigators on selecting, implementing, and interpreting MCI ascertainment approaches using routinely collected health‐care data.

**TABLE 3:** Practical guidance for using routinely collected health‐care data to measure MCI in embedded pragmatic clinical trials.

**Align MCI measurement with the role of MCI in ePCT design**
1. Select MCI ascertainment methods based on whether MCI is used for eligibility screening or outcome ascertainment.
2. For eligibility, prioritize sensitivity when confirmatory clinical assessment is feasible; when confirmatory assessment is impractical, greater specificity and PPV are prioritized.
3. For outcomes, prioritize specificity, clinical interpretability, and consistent ascertainment across intervention and control groups to minimize differential misclassification.
**Choose approaches appropriate for the study objective**
4. Prefer clinical criteria‐based approaches when feasible because they better approximate the clinical construct of MCI.
5. Use prediction‐based approaches primarily for screening or risk stratification (e.g., enrolling higher risk patients) rather than endpoint definition.
6. Do not rely solely on documentation‐based approaches for eligibility, as substantial under‐ascertainment may reduce sample size, representativeness, and generalizability.
7. When documentation‐based approaches (e.g., ICD diagnosis codes) are used, define the outcome explicitly as “documented MCI.”
8. When feasible, consider a two‐stage eligibility strategy using routine data screening followed by targeted clinical confirmation.
**Strengthen ascertainment and interpretation**
9. Use complementary data sources (e.g., MDS, OASIS) when available to improve capture of cognitive and functional information.
10. Consider using NLP or related methods to extract cognitive test results, functional status, and clinical impressions from unstructured EHR notes.
11. Use tiered definitions (e.g., definite, probable, possible MCI) to support sensitivity analyses across levels of diagnostic certainty.
12. Consider local pilot validation or targeted chart review to assess whether ascertainment methods identify the intended population consistently across participating study settings.
13. Prespecify alternative definitions and conduct sensitivity analyses to assess robustness to misclassification.
**Address disparities and sources of bias**
14. Consider whether MCI ascertainment may differ across populations and care settings because of differences in health‐care access, language, and documentation practices, potentially affecting representativeness and interpretation of trial findings.
15. When using NLP‐, LLM‐, or machine learning‐based methods, consider whether performance may vary across health‐care systems and patient populations.
**Report methods transparently**
16. Clearly define the construct being measured (e.g., “documented MCI,” “clinical criteria‐based MCI,” or “predicted risk of MCI”).
17. Report data sources, code lists, algorithms, observation windows, inclusion/exclusion criteria, and methods used to extract unstructured data.
18. Report validation performance characteristics (e.g., sensitivity, specificity, PPV, NPV, AUROC) when available.

Abbreviations: AUROC, area under the receiver operating characteristic curve; EHR, electronic health records; ePCT, embedded pragmatic clinical trial; ICD, International Classification of Diseases; LLM, large language model; MCI, mild cognitive impairment; MDS, Minimum Data Set; NLP, natural language processing; NPV, negative predictive value; OASIS, Outcome and Assessment Information Set; PPV, positive predictive value.

## 6 IMPLICATIONS OF HEALTH‐CARE DISPARITIES FOR MCI ASCERTAINMENT IN ePCTs

Health‐care disparities can affect all three approaches to MCI ascertainment described above. Documentation‐based approaches depend on clinical recognition and diagnosis, clinical criteria‐based approaches depend on access to cognitive assessment and documentation, and prediction‐based approaches may inherit biases present in the underlying data. As a result, MCI ascertainment may differ systematically across populations and care settings.

Differential access to diagnostic evaluation and specialty care contributes to delayed recognition among racial and ethnic minority groups and those with lower socioeconomic status.^48^, ^49^, ^50^, ^51^, ^52^ Commonly used cognitive screening instruments may perform differently across racial, educational, and linguistic groups^53^, ^54^, ^55^ and have higher false‐negative rates compared to neuropsychological and biomarker testing.^56^, ^57^ In addition, documentation of MCI is less likely among underserved populations because of differences in health‐care access, clinician practices, coding, and availability of culturally and linguistically appropriate assessments.^12^, ^58^, ^59^, ^60^ Stigma, medical mistrust, cultural differences in the attribution of cognitive symptoms, and availability of informants may further affect ascertainment.

These disparities may result in differential misclassification of MCI and affect both the representativeness of study populations and interpretation of treatment effects. Investigators should consider whether ascertainment methods perform differently across populations and care settings,^61^ conduct sensitivity analyses, and validation algorithms in diverse populations.^19^, ^20^ When using natural language processing and large language models, attention should be paid to potential algorithmic bias that may replicate or amplify existing disparities.^62^, ^63^

## 7 FUTURE DIRECTIONS

Advancing the measurement of MCI in routinely collected health‐care data will require coordinated progress in phenotype development, data infrastructure, and implementation. Rather than relying on diagnosis codes alone, future work should focus on reproducible, clinically grounded phenotypes that can be applied consistently across studies and health systems, enabling more valid and generalizable use of routinely collected health‐care data in ePCTs.

First, efforts should prioritize the development and validation of clinical criteria‐based phenotypes that more fully capture the diagnostic construct of MCI, including cognitive impairment, functional status, and exclusion of competing conditions. Most existing approaches capture only partial domains; establishing standardized and reproducible MCI approaches for routinely collected health‐care data can improve comparability across studies and health‐care systems.

Second, there is a need for more granular and longitudinal measures of MCI, moving beyond binary classification to capture progression to dementia over time. Such measures are critical for evaluating intervention effects and aligning outcomes with clinically meaningful changes.

Third, systematic evaluation of measurement performance across health‐care systems, care settings, and patient populations is essential. Differences in access to care, specialty evaluation, and documentation practices may lead to differential ascertainment. Future work should prioritize validation in diverse populations and assessment of potential bias across sociodemographic groups.

Fourth, progress will depend on strengthening data infrastructure and standardization in routine care. Structured capture of cognitive testing, functional status, and patient‐reported outcomes remains limited within EHR systems. Expanding structured data elements and improving interoperability, and leveraging natural language processing, large language models, and multimodal data sources may enhance MCI ascertainment.^64^ Integration with frailty measures and digital biomarkers, wearables, and mobile technologies, offers opportunities to capture cognitive and functional changes beyond clinical encounters.

Finally, evolving clinical practice and policy initiatives are likely to increase the availability of cognitive data in routine care. The introduction of anti‐amyloid monoclonal antibody therapies,^9^, ^10^ which require formal cognitive assessment for treatment eligibility, and initiatives such as the Centers for Medicare and Medicaid Services Age‐Friendly Hospital Measure^65^ are expected to expand cognitive screening and documentation. These developments may create new opportunities to improve the measurement of MCI and accelerate implementation of scalable approaches for research and clinical care.

## 8 CONCLUSIONS

Accurate measurement of MCI using routinely collected health‐care data is essential for ePCTs but remains challenging because key diagnostic elements are incompletely captured and clinical recognition and documentation vary across health‐care settings. Documentation‐based, clinical criteria‐based, and prediction‐based approaches measure different constructs and are not interchangeable.

Clinical criteria‐based approaches should be prioritized whenever feasible because they most closely approximate the diagnostic construct of MCI. Documentation‐based approaches may serve as complementary measures of clinician‐recognized MCI, whereas prediction‐based approaches are best suited for screening, risk stratification, and enrichment rather than outcome ascertainment.

Investigators should align ascertainment methods with the role of MCI in ePCTs, evaluate robustness to misclassification, and consider the impact of health‐care disparities on measurement performance. Continued advances in phenotype development, data standardization, and validation will be critical to enable more accurate and scalable MCI ascertainment in ePCTs.

## CONFLICT OF INTEREST STATEMENT

D.H.K. received personal fees from Pfizer and Rainfall Health for unrelated work. D.H.K., D.H.L., E.M.W., and H.X. received grants from the National Institutes of Health (paid to institution). E.M.W. also received grants from the Centers for Disease Control and Prevention and the Warren Alpert Foundation (paid to institution). J.L.L. received consulting fees from GSK (paid to institution) and honoraria from Regeneron. The other authors report no additional disclosures. Author disclosures are available in the Supporting Information.

## Supporting information



Supporting Information

## References

[1] Albert MS , DeKosky ST , Dickson D , et al. The diagnosis of mild cognitive impairment due to Alzheimer's disease: recommendations from the National Institute on Aging‐Alzheimer's Association workgroups on diagnostic guidelines for Alzheimer's disease. Alzheimers Dement. 2011;7(3):270‐279. doi:10.1016/j.jalz.2011.03.008 10.1016/j.jalz.2011.03.008 PMC3312027 21514249

[2] Petersen RC , Smith GE , Waring SC , Ivnik RJ , Tangalos EG , Kokmen E . Mild cognitive impairment: clinical characterization and outcome. Arch Neurol. 1999;56(3):303‐308. doi:10.1001/archneur.56.3.303 10.1001/archneur.56.3.303 10190820

[3] American Psychiatric Association . Diagnostic and statistical manual of mental disorders American Psychiatric Association 5th ed. text rev. 2022

[4] Petersen RC , Lopez O , Armstrong MJ , et al. Practice guideline update summary: mild cognitive impairment [RETIRED]: report of the Guideline Development, Dissemination, and Implementation Subcommittee of the American Academy of Neurology. Neurology. 2018;90(3):126‐135. doi:10.1212/WNL.0000000000004826 10.1212/WNL.0000000000004826 PMC5772157 29282327

[5] De Vriendt P , Gorus E , Cornelis E , Bautmans I , Petrovic M , Mets T . The advanced activities of daily living: a tool allowing the evaluation of subtle functional decline in mild cognitive impairment. J Nutr Health Aging. 2013;17(1):64‐71. doi:10.1007/s12603‐012‐0381‐9 10.1007/s12603-012-0381-9 PMC12879642 23299382

[6] Callahan KE , Lovato JF , Miller ME , Easterling D , Snitz B , Williamson JD . Associations Between Mild Cognitive Impairment and Hospitalization and Readmission. J Am Geriatr Soc. 2015;63(9):1880‐1885. doi:10.1111/jgs.13593 10.1111/jgs.13593 PMC4809245 26313420

[7] Yaffe K , Petersen RC , Lindquist K , Kramer J , Miller B . Subtype of mild cognitive impairment and progression to dementia and death. Dement Geriatr Cogn Disord. 2006;22(4):312‐319. doi:10.1159/000095427 10.1159/000095427 16940725

[8] Frech FH , Li G , Juday TR , et al. Real‐world health care costs and resource utilization associated with mild cognitive impairment in the United States: a retrospective cohort study of commercial and Medicare data. J Manag Care Spec Pharm. 2025;31(8):782‐794. doi:10.18553/jmcp.2025.31.8.782 10.18553/jmcp.2025.31.8.782 PMC12288724 40704847

[9] Sims JR , Zimmer JA , Evans CD , et al. Donanemab in Early Symptomatic Alzheimer Disease: the TRAILBLAZER‐ALZ 2 Randomized Clinical Trial. JAMA. 2023;330(6):512‐527. doi:10.1001/jama.2023.13239 10.1001/jama.2023.13239 PMC10352931 37459141

[10] van Dyck CH , Swanson CJ , Aisen P , et al. Lecanemab in Early Alzheimer's Disease. N Engl J Med. 2023;388(1):9‐21. doi:10.1056/NEJMoa2212948 10.1056/NEJMoa2212948 36449413

[11] Aguilar BJ , Miller D , Jasuja G , et al. Rule‐Based Identification of Individuals with Mild Cognitive Impairment or Alzheimer's Disease Using Clinical Notes from the United States Veterans Affairs Healthcare System. Neurol Ther. 2023;12(6):2067‐2078. doi:10.1007/s40120‐023‐00540‐2 10.1007/s40120-023-00540-2 PMC10630261 37747662

[12] Mattke S , Jun H , Chen E , Liu Y , Becker A , Wallick C . Expected and diagnosed rates of mild cognitive impairment and dementia in the U.S. Medicare population: observational analysis. Alzheimers Res Ther. 2023;15(1):128. doi:10.1186/s13195‐023‐01272‐z 10.1186/s13195-023-01272-z PMC10362635 37481563

[13] Liu Y , Jun H , Becker A , Wallick C , Mattke S . Detection Rates of Mild Cognitive Impairment in Primary Care for the United States Medicare Population. J Prev Alzheimers Dis. 2024;11(1):7‐12. doi:10.14283/jpad.2023.131 10.14283/jpad.2023.131 PMC10995024 38230712

[14] Chubak J , Pocobelli G , Weiss NS . Tradeoffs between accuracy measures for electronic health care data algorithms. J Clin Epidemiol. 2012;65(3):343‐349. e342. doi:10.1016/j.jclinepi.2011.09.002 10.1016/j.jclinepi.2011.09.002 PMC3264740 22197520

[15] Bacigalupo I , Lombardo FL , Bargagli AM , et al. Identification of dementia and MCI cases in health information systems: an Italian validation study. Alzheimers Dement (N Y). 2022;8(1):e12327. doi:10.1002/trc2.12327 10.1002/trc2.12327 PMC9617569 36320346

[16] McCarthy EP , Chang CH , Tilton N , Kabeto MU , Langa KM , Bynum JPW . Validation of Claims Algorithms to Identify Alzheimer's Disease and Related Dementias. J Gerontol A Biol Sci Med Sci. 2022;77(6):1261‐1271. doi:10.1093/gerona/glab373 10.1093/gerona/glab373 PMC9159657 34919686

[17] Carrell DS , Floyd JS , Gruber S , et al. A general framework for developing computable clinical phenotype algorithms. J Am Med Inform Assoc. 2024;31(8):1785‐1796. doi:10.1093/jamia/ocae121 10.1093/jamia/ocae121 PMC11258420 38748991

[18] Li G , Cobb B , Nelson TM , et al. Risk prediction of mild cognitive impairment using electronic health record data. Alzheimers Dement (Amst). 2025;17(4):e70209. doi:10.1002/dad2.70209 10.1002/dad2.70209 PMC12644922 41306115

[19] Wei R , Buss SS , Milde R , et al. Automated phenotyping of mild cognitive impairment and Alzheimer's disease and related dementias using electronic health records. Int J Med Inform. 2025;200:105917. doi:10.1016/j.ijmedinf.2025.105917 10.1016/j.ijmedinf.2025.105917 PMC13719700 40222334

[20] Shankar R , Bundele A , Mukhopadhyay A . Natural language processing of electronic health records for early detection of cognitive decline: a systematic review. NPJ Digit Med. 2025;8(1):133. doi:10.1038/s41746‐025‐01527‐z 10.1038/s41746-025-01527-z PMC11873039 40025194

[21] Wang KY , Mahesri M , Novoa‐Laurentiev J , et al. Extracting Cognitive Impairment Assessment Information From Unstructured Notes in Electronic Health Records Using Natural Language Processing Tools: validation with Clinical Assessment Data. Clin Epidemiol. 2025;17:353‐365. doi:10.2147/CLEP.S504259 10.2147/CLEP.S504259 PMC12009745 40260424

[22] Laurentiev J , Kim DH , Mahesri M , et al. Identifying Functional Status Impairment in People Living With Dementia Through Natural Language Processing of Clinical Documents: cross‐Sectional Study. J Med Internet Res. 2024;26:e47739. doi:10.2196/47739 10.2196/47739 PMC10900085 38349732

[23] Kistler CE , Lynch ME , Lin FC , Yang Y , Frazier W , Hanson LC . An Electronic Health Record Algorithm's Performance to Identify Cognitive Impairment in Primary Care. J Am Med Dir Assoc. 2026;27(1):105949. doi:10.1016/j.jamda.2025.105949 10.1016/j.jamda.2025.105949 PMC13290007 41135594

[24] Maclagan LC , Abdalla M , Harris DA , et al. Can Patients with Dementia Be Identified in Primary Care Electronic Medical Records Using Natural Language Processing?. J Healthc Inform Res. 2023;7(1):42‐58. doi:10.1007/s41666‐023‐00125‐6 10.1007/s41666-023-00125-6 PMC9995630 36910911

[25] Ciesielska N , Sokolowski R , Mazur E , Podhorecka M , Polak‐Szabela A , Kedziora‐Kornatowska K . Is the Montreal Cognitive Assessment (MoCA) test better suited than the Mini‐Mental State Examination (MMSE) in mild cognitive impairment (MCI) detection among people aged over 60? Meta‐analysis. Psychiatr Pol. 2016;50(5):1039‐1052. doi:10.12740/PP/45368 10.12740/PP/45368 27992895

[26] Floden DP , Hogue O , Saxena SA , et al. Automated identification of older adults at risk for cognitive decline. Alzheimers Dement (Amst). 2025;17(2):e70136. doi:10.1002/dad2.70136 10.1002/dad2.70136 PMC12162263 40520422

[27] Penfold RB , Carrell DS , Cronkite DJ , et al. Development of a machine learning model to predict mild cognitive impairment using natural language processing in the absence of screening. BMC Med Inform Decis Mak. 2022;22(1):129. doi:10.1186/s12911‐022‐01864‐z 10.1186/s12911-022-01864-z PMC9097352 35549702

[28] Wang L , Laurentiev J , Yang J , et al. Development and Validation of a Deep Learning Model for Earlier Detection of Cognitive Decline From Clinical Notes in Electronic Health Records. JAMA Netw Open. 2021;4(11):e2135174. doi:10.1001/jamanetworkopen.2021.35174 10.1001/jamanetworkopen.2021.35174 PMC8603078 34792589

[29] Du X , Novoa‐Laurentiev J , Plasek JM , et al. Enhancing early detection of cognitive decline in the elderly: a comparative study utilizing large language models in clinical notes. EBioMedicine. 2024;109:105401. doi:10.1016/j.ebiom.2024.105401 10.1016/j.ebiom.2024.105401 PMC11663780 39396423

[30] Leng Y , He Y , Amini S , et al. A GPT‐4o‐powered framework for identifying cognitive impairment stages in electronic health records. NPJ Digit Med. 2025;8(1):401. doi:10.1038/s41746‐025‐01834‐5 10.1038/s41746-025-01834-5 PMC12229571 40610683

[31] Mitchell SL , Mor V , Harrison J , McCarthy EP . Embedded Pragmatic Trials in Dementia Care: realizing the Vision of the NIA IMPACT Collaboratory. J Am Geriatr Soc. 2020;68(Suppl 2):S1‐S7. doi:10.1111/jgs.16621 10.1111/jgs.16621 PMC8020214 32589280

[32] Wolff JL , DesRoches CM , Amjad H , et al. Catalyzing dementia care through the learning health system and consumer health information technology. Alzheimers Dement. 2023;19(5):2197‐2207. doi:10.1002/alz.12918 10.1002/alz.12918 PMC10182243 36648146

[33] Barnes DE , Zhou J , Walker RL , et al. Development and Validation of eRADAR: a Tool Using EHR Data to Detect Unrecognized Dementia. J Am Geriatr Soc. 2020;68(1):103‐111. doi:10.1111/jgs.16182 10.1111/jgs.16182 PMC7094818 31612463

[34] Creavin ST , Noel‐Storr AH , Langdon RJ , et al. Clinical judgement by primary care physicians for the diagnosis of all‐cause dementia or cognitive impairment in symptomatic people. Cochrane Database Syst Rev. 2022;6(6):CD012558. doi:10.1002/14651858.CD012558.pub2 10.1002/14651858.CD012558.pub2 PMC9202995 35709018

[35] O'Brien K , Burke R , Karlawish J . A Roadmap for Modifying Clinician Behavior to Improve the Detection of Cognitive Impairment. J Gen Intern Med. 2023;38(2):508‐512. doi:10.1007/s11606‐022‐07824‐7 10.1007/s11606-022-07824-7 PMC9905516 36163531

[36] Dubois B , Padovani A , Scheltens P , Rossi A , Dell'Agnello G . Timely Diagnosis for Alzheimer's Disease: a Literature Review on Benefits and Challenges. J Alzheimers Dis. 2016;49(3):617‐631. doi:10.3233/JAD‐150692 10.3233/JAD-150692 PMC4927869 26484931

[37] Shi S , Largent EA , McCreedy E , Mitchell SL . Design Considerations for Embedded Pragmatic Clinical Trials of Advance Care Planning Interventions for Persons Living With Dementia. J Pain Symptom Manage. 2023;65(2):e155‐e163. doi:10.1016/j.jpainsymman.2022.11.009 10.1016/j.jpainsymman.2022.11.009 PMC9875559 36423803

[38] Tuzzio L , Hanson LR , Reuben DB , et al. Transforming Dementia Care Through Pragmatic Clinical Trials Embedded in Learning Healthcare Systems. J Am Geriatr Soc. 2020;68(Suppl 2):S43‐S48. doi:10.1111/jgs.16629 10.1111/jgs.16629 PMC7419336 32589283

[39] Bynum JPW , Dorr DA , Lima J , et al. Using Healthcare Data in Embedded Pragmatic Clinical Trials among People Living with Dementia and Their Caregivers: state of the Art. J Am Geriatr Soc. 2020;68(Suppl 2):S49‐S54. doi:10.1111/jgs.16617 10.1111/jgs.16617 PMC7924260 32589274

[40] Pike KJ , Fazio S , Bynum JPW , Travison TG , Wendler D , Mor V . Resources, methods, and data infrastructure to promote research in dementia care, caregiving, and services. J Am Geriatr Soc. 2021;69(7):1793‐1800. doi:10.1111/jgs.17339 10.1111/jgs.17339 PMC8351358 34245589

[41] Cox CG , Brush BL , Kobayashi LC , Roberts JS . Determinants of dementia diagnosis in U.S. primary care in the past decade: a scoping review. J Prev Alzheimers Dis. 2025;12(2):100035. doi:10.1016/j.tjpad.2024.100035 10.1016/j.tjpad.2024.100035 PMC12184027 39863322

[42] Force U , Owens DK , Davidson KW , et al. Screening for Cognitive Impairment in Older Adults: uS Preventive Services Task Force Recommendation Statement. JAMA. 2020;323(8):757‐763. doi:10.1001/jama.2020.0435 10.1001/jama.2020.0435 32096858

[43] McCreedy E , Gilmore‐Bykovskyi A , Dorr DA , et al. Barriers to identifying residents with dementia for embedded pragmatic trials: a call to action. J Am Geriatr Soc. 2022;70(2):638‐641. doi:10.1111/jgs.17539 10.1111/jgs.17539 PMC8821246 34727369

[44] Hanson LC , Bennett AV , Jonsson M , et al. Selecting Outcomes to Ensure Pragmatic Trials Are Relevant to People Living with Dementia. J Am Geriatr Soc. 2020;68(Suppl 2):S55‐S61. doi:10.1111/jgs.16619 10.1111/jgs.16619 PMC7469944 32589279

[45] Hanson LC , Wessell K , Meeks N , et al. Selecting outcomes for pragmatic clinical trials in dementia care: the IMPACT Collaboratory iLibrary. J Am Geriatr Soc. 2024;72(2):529‐535. doi:10.1111/jgs.18644 10.1111/jgs.18644 PMC10922084 37916447

[46] Gabb VG , Harding S , Lemke T , et al. Outcomes reported in interventional studies for mild cognitive impairment: a scoping umbrella review to inform core outcome set development. Alzheimers Dement. 2026;22(9):e71800. doi:10.1002/alz.71800 10.1002/alz.71800 PMC13546403 42701205

[47] Walling AM , Pevnick J , Bennett AV , Vydiswaran VGV , Ritchie CS . Dementia and electronic health record phenotypes: a scoping review of available phenotypes and opportunities for future research. J Am Med Inform Assoc. 2023;30(7):1333‐1348. doi:10.1093/jamia/ocad086 10.1093/jamia/ocad086 PMC10280354 37252836

[48] Gleason CE , Norton D , Zuelsdorff M , et al. Association between enrollment factors and incident cognitive impairment in Blacks and Whites: data from the Alzheimer's Disease Center. Alzheimers Dement. 2019;15(12):1533‐1545. doi:10.1016/j.jalz.2019.07.015 10.1016/j.jalz.2019.07.015 PMC6925619 31601516

[49] Lennon JC , Aita SL , Bene VAD , et al. Black and White individuals differ in dementia prevalence, risk factors, and symptomatic presentation. Alzheimers Dement. 2022;18(8):1461‐1471. doi:10.1002/alz.12509 10.1002/alz.12509 PMC9160212 34854531

[50] Mace RA , Mansbach WE . Disparities in Dementia Occurrence and Rate of Cognitive Decline Between African American and White Long‐Term Care Residents. Res Gerontol Nurs. 2019;13:1‐7. doi:10.3928/19404921‐20191030‐01 10.3928/19404921-20191030-01 31697390

[51] Ramirez Gomez L , Jain FA , D'Orazio LM . Assessment of the Hispanic Cognitively Impaired Elderly Patient. Neurol Clin. 2017;35(2):207‐229. doi:10.1016/j.ncl.2017.01.003 10.1016/j.ncl.2017.01.003 28410657

[52] Rawat P , Sehar U , Bisht J , Reddy AP , Reddy PH . Alzheimer's disease and Alzheimer's disease‐related dementias in Hispanics: identifying influential factors and supporting caregivers. Ageing Res Rev. 2024;93:102178. doi:10.1016/j.arr.2023.102178 10.1016/j.arr.2023.102178 PMC10807242 38154509

[53] Espino DV , Lichtenstein MJ , Palmer RF , Hazuda HP . Ethnic differences in mini‐mental state examination (MMSE) scores: where you live makes a difference. J Am Geriatr Soc. 2001;49(5):538‐548. doi:10.1046/j.1532‐5415.2001.49111.x 10.1046/j.1532-5415.2001.49111.x 11380745

[54] Sachs BC , Chelune GJ , Rapp SR , et al. Robust demographically‐adjusted normative data for the Montreal Cognitive Assessment (MoCA): results from the systolic blood pressure intervention trial. Clin Neuropsychol. 2022;36(8):2237‐2259. doi:10.1080/13854046.2021.1967450 10.1080/13854046.2021.1967450 PMC8885785 34470584

[55] Pilonieta G , Geldmacher DS . Differential cognitive and functional performance in racial and ethnic groups when stratified by consensus diagnosis: findings from the NACC dataset. Alzheimer's Dement. 2023;19:e073708. doi:10.1002/alz.073708

[56] Edmonds EC , Delano‐Wood L , Jak AJ , et al. “Missed” Mild Cognitive Impairment: high False‐Negative Error Rate Based on Conventional Diagnostic Criteria. J Alzheimers Dis. 2016;52(2):685‐691. doi:10.3233/JAD‐150986 10.3233/JAD-150986 PMC4879874 27031477

[57] Drabo EF , Barthold D , Joyce G , Ferido P , Chang Chui H , Zissimopoulos J . Longitudinal analysis of dementia diagnosis and specialty care among racially diverse Medicare beneficiaries. Alzheimers Dement. 2019;15(11):1402‐1411. doi:10.1016/j.jalz.2019.07.005 10.1016/j.jalz.2019.07.005 PMC6874742 31494079

[58] Mukadam N , Cooper C , Livingston G . A systematic review of ethnicity and pathways to care in dementia. Int J Geriatr Psychiatry. 2011;26(1):12‐20. doi:10.1002/gps.2484 10.1002/gps.2484 21157846

[59] Maher C , Calia C . The effect of illiteracy on performance in screening tools for dementia: a meta‐analysis. J Clin Exp Neuropsychol. 2021;43(10):945‐966. doi:10.1080/13803395.2022.2040433 10.1080/13803395.2022.2040433 35200100

[60] Stimmel MB , Orkaby AR , Ayers E , Verghese J , Nsubayi CK , Weiss EF . Is the Montreal cognitive assessment culturally valid in a diverse geriatric primary care setting? Lessons from the Bronx. J Am Geriatr Soc. 2024;72(3):850‐857. doi:10.1111/jgs.18705 10.1111/jgs.18705 PMC10947962 38196332

[61] Nicholls SG , AA Al‐Jaishi , Niznick H , et al. Health equity considerations in pragmatic trials in Alzheimer's and dementia disease: results from a methodological review. Alzheimers Dement (Amst). 2023;15(1):e12392. doi:10.1002/dad2.12392 10.1002/dad2.12392 PMC9899766 36777091

[62] Chen IY , Joshi S , Ghassemi M . Treating health disparities with artificial intelligence. Nat Med. 2020;26(1):16‐17. doi:10.1038/s41591‐019‐0649‐2 10.1038/s41591-019-0649-2 31932779

[63] Obermeyer Z , Powers B , Vogeli C , Mullainathan S . Dissecting racial bias in an algorithm used to manage the health of populations. Science. 2019;366(6464):447‐453. doi:10.1126/science.aax2342 10.1126/science.aax2342 31649194

[64] Lyketsos CG , Roberts SB , Swift EK , et al. Standardizing Electronic Health Record Data on AD/ADRD to Accelerate Health Equity in Prevention, Detection, and Treatment. J Prev Alzheimers Dis. 2022;9(3):556‐560. doi:10.14283/jpad.2022.47 10.14283/jpad.2022.47 35841257

[65] Centers for Medicare & Medicaid Services . Medicare and Medicaid programs and the Children's Health Insurance Program; hospital inpatient prospective payment systems for acute care hospitals and the long‐term care hospital prospective payment system and policy changes and fiscal year 2025 rates. Fed Regist. 2024;89(167):68994‐69464.

